# Perspective: The Future of the Southern Resident Killer Whales Depends on Interactions With Other Killer Whale Populations

**DOI:** 10.1002/ece3.73205

**Published:** 2026-03-05

**Authors:** Michael J. Ford, Eric J. Ward, Marty Kardos, Kim M. Parsons, Candice Emmons, M. Bradley Hanson

**Affiliations:** ^1^ Conservation Biology Division, Northwest Fisheries Science Center National Oceanic and Atmospheric Administration Seattle Washington USA

**Keywords:** Chinook salmon, competition, endangered species, inbreeding, interbreeding, orca

## Abstract

Ecological and genetic interactions among conspecific populations play an important role in population viability, but these interactions are not always fully considered in strategies to recover endangered taxa. Southern Resident killer whales are a high‐profile population listed as endangered by both the United States and Canada. Risks to the population are well known, and include insufficient prey, inbreeding depression, disturbance, and environmental contaminants. Here, we argue that a fifth factor—interactions with other sympatric killer whale populations—plays an underappreciated role in the population's current and potential status. Based on studies conducted over the past two decades, we illustrate that consumption of shared prey, behavioral interactions in shared habitat, and shared DNA through potential interbreeding with other populations will strongly influence the future trajectory of the Southern Resident killer whales.

## Graphical abstract

The Southern Resident Killer Whales are a high‐profile population listed as endangered in the United States and Canada. Risks to the population are well known, and include insufficient prey, inbreeding depression, disturbance, and environmental contaminants. We argue that a fifth factor—interactions with other sympatric killer whale populations—plays an underappreciated role in the population's current and potential status.

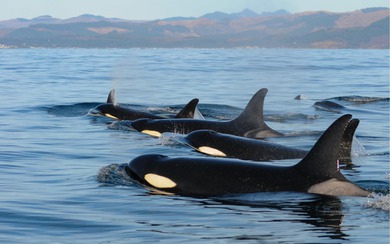

## 1 Introduction

**FIGURE 1:**
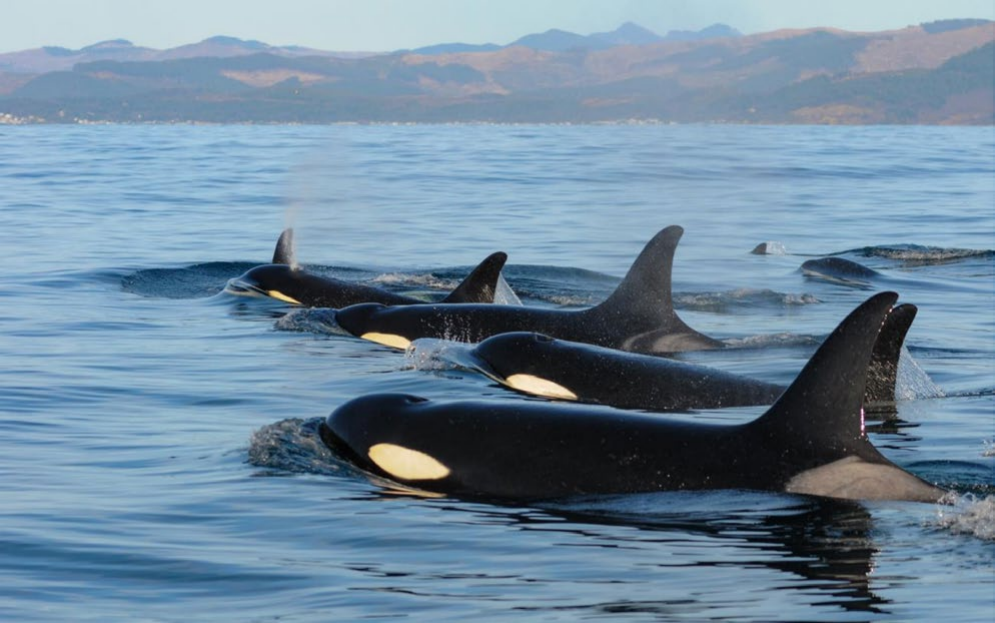
A group of Southern Resident killer whales off the coast of Washington in March 2013. Photo credit: NOAA Fisheries, taken under NOAA Research Permit #16163.

The Southern Resident killer whales (SRKW; *Orcinus orca ater* ) are a well‐known population that has been the subject of scientific and popular interest since the late 1960s (Ford et al. 2000). They live primarily in the coastal waters of Washington and southern British Columbia (Figure 1), occasionally ranging to California and southeast Alaska (NMFS 2021a). They have been protected as endangered in both the United States and Canada since the early 2000s (NMFS 2005; Fisheries and Ocean Canada 2011).

At the time of their listing in 2005, four primary risk factors were cited: insufficient salmon prey, physical and acoustic disturbance from vessels, health effects from environmental contaminants, and the small, genetically isolated nature of the population (NMFS 2008). Two decades of research have refined our understanding of these factors and confirmed that they all remain as threats (NMFS 2021b).

Despite scientific progress and protection measures implemented over the past 20 years, the SRKW have continued to decline, from an estimated minimum of 140 individuals historically, to 89 individuals when they were listed under the ESA in 2005 (NMFS 2008), to 74 at the time of the most recent census in 2025 (CWR 2026). The decline after 2005 occurred despite efforts to increase prey, decrease disturbance, and reduce contaminant inputs (NMFS 2021b). Under current conditions, the population is predicted to decline to fewer than 50 individuals within several decades (NMFS 2021b; Kardos et al. 2023; Williams et al. 2024). These predictions have led to new proposals to further reduce risks to the SRKW population, including additional prey increases through harvest reductions (Graham 2023), increasing hatchery releases (NMFS 2024), and new, large‐scale habitat recovery efforts, such as major dam removals (Connelly 2018), along with further actions to reduce vessel impacts (WDFW 2024). More prey, less disturbance, and continued mitigation of environmental threats will undoubtedly benefit SRKW. Our purpose here, however, is to bring attention to another important element that we believe has been underappreciated in SRKW population dynamics—interactions between SRKW and other killer whale populations.

Research over the past two decades shows that ecological and genetic interactions between SRKW and other killer whale populations may well determine whether SRKW persist or not. Below, we explain why we think this is true and recommend research that will further characterize these important interactions. These ideas are not all new, and many can be found in the discussion sections of papers published over the past 20 years. Here, we summarize this information in one place to reinforce the importance of population interactions for the recovery of the SRKW population.

## 2 A Brief History of Northeast Pacific Killer Whales

Killer whales are found in all the world's oceans, divided into many local populations (Heyning and Dahlheim 1988; Ford et al. 1998). These populations are ecologically, genetically, and behaviorally diverse, including three recognized subspecies (Reeves et al. 2004; NMFS 2005; Costa 2024). Due to this diversity, there have been proposals to split *O. orca* into multiple species (Morin et al. 2010, 2024), but only a single species is currently recognized (Society for Marine Mammalogy 2025).

Within the NE Pacific, the National Marine Fisheries Service (NMFS) recognizes seven killer whale populations (NMFS 2025). The total number is likely larger, based on genetic structuring that is not yet reflected in stock designations (Parsons et al. 2013). The populations are grouped into three ‘ecotypes’ (also recognized as subspecies—Society for Marine Mammalogy 2025) based on diet, genetics, morphology and behavior (Bigg et al. 1987, 1990; Ford et al. 2000): mammal‐eating Bigg's killer whales (*O. o. rectipinnus*), and fish‐eating “resident” (*O. o. ater*) and “offshore” (*O. o. orca*) killer whales (Dahlheim et al. 2008). Multiple populations of each ecotype can be found around the Pacific Rim (Krahn et al. 2002; Parsons et al. 2013; Mclnnes et al. 2024; NMFS 2025).

Globally, killer whale population structure is influenced by a history of population splitting followed by episodic gene flow (Foote 2012; Hoelzel and Moura 2015; Foote et al. 2016, 2019; Foote and Morin 2016). Within the NE Pacific, these splitting events have occurred at widely varying times. The deepest divergence is between the resident and Bigg's lineages, which diverged prior to their colonization of the Pacific Ocean, estimated to be anywhere from < 10,000 to > 300,000 years ago (Hoelzel et al. 2007; Morin et al. 2015; Foote and Morin 2016; Foote et al. 2019). The divergence between offshores and residents is estimated to be < 100,000 years ago (Morin et al. 2015). Populations within ecotypes are more recently diverged, estimated to range from a few hundred to a few thousand years ago (Hoelzel et al. 2007; Kardos et al. 2023).

In addition to splitting that leads to ecological, genetic and behavioral divergence, there is evidence for widespread gene flow among populations. In the NE Pacific, estimates range from < 1 or 2 exchanges per generation among ecotypes, to > 20 exchanges per generation among some putative populations within ecotypes (Hoelzel et al. 2007). Globally, killer whale genomes have been found to have patterns of variation that suggest a history of episodic gene flow events, even among ecologically divergent populations, although these may have occurred via intermediate populations (Foote et al. 2019). A paternity analysis has also identified progeny from matings between populations in the NE Pacific, indicating some recent gene flow (Pilot et al. 2010).

In this context, SRKW are clearly one of many killer whale populations in the NE Pacific, with varying degrees of ecological and genetic interactions. The implications of this for SRKW conservation will be developed below.

## 3 How Interactions With Other Orca Populations Influence SRKW

SRKW can potentially interact with other NE Pacific populations in at least three ways: consumption of shared prey; behavioral interactions in shared habitat; and shared DNA through interbreeding. We discuss each of these in turn below.

### 3.1 Consumption of Shared Prey

SRKWs eat mostly salmon, along with a variety of other fish species (Ford et al. 1998, 2016; Ford and Ellis 2006; Hanson et al. 2010, 2021). Based on genetic analyses of feces and prey remains, Chinook salmon ( *Oncorhynchus tshawytscha* ), primarily from rivers in the Pacific Northwest and southern British Columbia, are the most common species in SRKW's diet (Ford and Ellis 2006; Hanson et al. 2021). After leaving their natal streams, Chinook migrate north in the ocean to feed and grow. During their return migration, the now‐large Chinook are preyed upon by SRKW and at least three other killer whale populations—the Northern Resident killer whales (NRKW), Southern Alaskan Resident killer whales (SARKW; Ford, Wright, et al. 2010; Ward et al. 2016; Chasco et al. 2017; Van Cise et al. 2024), and offshores (Dahlheim et al. 2008).

Insufficient Chinook prey has long been identified as a risk factor for SRKW (NMFS 2008). Evidence for this includes the relative rarity of Chinook and temporal correlations between Chinook abundance and SRKW survival (Ford, Ellis, et al. 2010; Ward et al. 2013; Nelson et al. 2024), fecundity (Ward et al. 2009), physiology (Ayres et al. 2012), and condition (Stewart et al. 2021).

What is less appreciated is that NRKW and SARKW also share some of these correlations, both with Chinook salmon and with SRKW (Ford, Ellis, et al. 2010; Ward et al. 2016). Annual birth rates in SRKW and SARKW, for example, are highly correlated (Ward et al. 2016). Similarly, most of the fish‐eating killer whale populations in the NE Pacific had low survival in the mid‐late 1990s, when Chinook salmon populations also declined. A plausible explanation for these patterns is that the populations share the same salmon prey, and variation in prey abundance affects all of these populations together (Ward et al. 2016). In other words, SRKW are likely competing for prey with other fish‐eating killer whale populations (Chasco et al. 2017; Hanson et al. 2021).

This competition has some important implications for SRKW recovery. While the SRKW population has remained small, NRKW and SARKW have increased 2‐to‐3‐fold since the mid‐1970s, and there are now more than 2000 fish‐eating killer whales in the NE Pacific (Carretta et al. 2020; Young et al. 2023) that are estimated to consume > 2 M adult Chinook annually (Chasco et al. 2017). Within NRKW, the subgroup (G clan) whose distribution most overlaps with SRKW's has increased from < 50 to about 100 individuals (Towers et al. 2020), an increase roughly proportional to the SRKW decline. Any increases in Chinook salmon abundance will be shared with other killer whale populations, along with other marine mammal populations, and thus may provide only marginal benefits to SRKW (Hilborn et al. 2012). It is therefore important to evaluate the effects of changes in prey abundance within the context of all fish‐eating killer whale populations, not just SRKW.

### 3.2 Use of Shared Habitat

**FIGURE 2:**
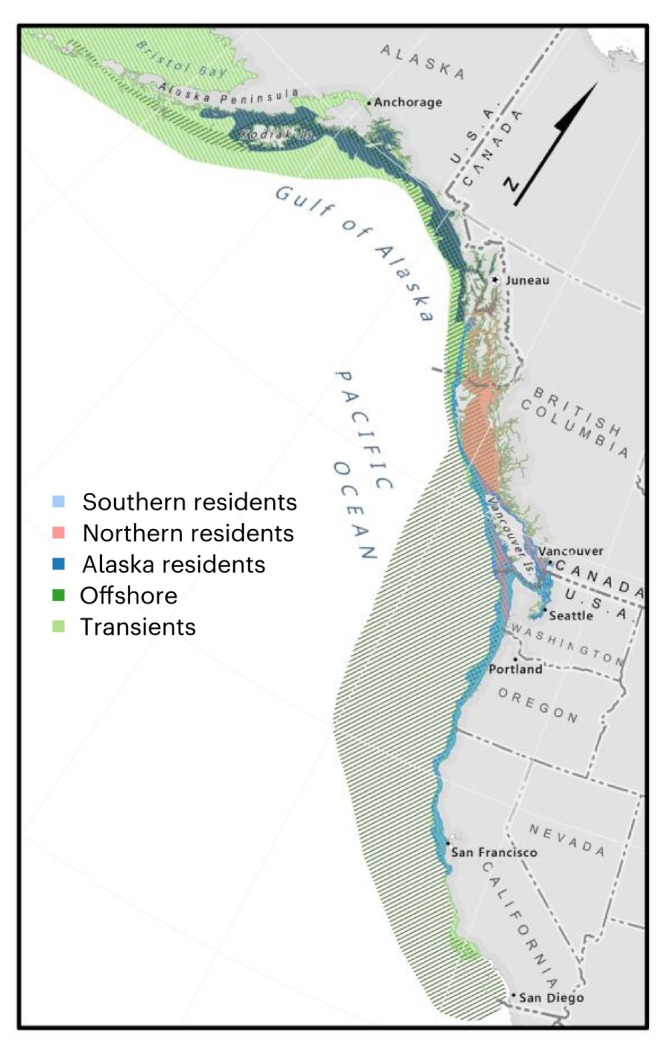
Range map of five NE Pacific killer whale populations. Cross hatching is used to facilitate visualization of overlap.

In addition to common prey, SRKW share their habitat with three other killer whale populations (Figure 2). Overlapping ranges allow for potential behavioral interactions, including direct competition for prey or space, displacement, or interbreeding. Our focus here is primarily on the potential for interactions between SRKW and the closely related NRKW, although interactions with other populations may also be important.

The ranges of SRKW and NRKW have long been known to partially overlap (Ford et al. 1998, 2000; Dahlheim et al. 2008; Figure 2). Recent research shows that the overlap and potential for interactions may be larger than previously realized and may be increasing in response to changing prey distributions. Acoustic monitoring off the Washington Coast demonstrates that both populations use this area, particularly in spring and late summer (Emmons et al. 2021). SRKW and NRKW (and Bigg's KW) also all use the northern Strait of Georgia extensively in winter (Pilkington et al. 2023). Both of these studies found fine‐scale temporal or spatial segregation, suggesting some degree of habitat partitioning. Nonetheless, individuals from each population were detected at the same locations on the same days, sometimes within hours of each other, indicating at least the potential for behavioral interactions.

The SRKW distribution has also changed in ways that may increase opportunities for interaction with NRKW and offshores. There is a declining trend in SRKW use of what was previously considered core summer habitat in the central Salish Sea (Ettinger et al. 2022). The trend is statistically associated with changes in the abundance and timing of Fraser River Chinook salmon, a key prey item. In addition, over roughly the same time period, Bigg's killer whales have increased their use of the Salish Sea, preying on increasing numbers of pinnipeds in the area (Houghton et al. 2015; Shields et al. 2018). The increase in Bigg's killer whales might increase SRKW prey by reducing pinniped predation on salmon, but may also negatively affect SRKW by displacing them from their core habitat. As SRKW spend less time in the central Salish Sea and more time in habitats shared with other fish‐eating killer whales, their chances of interaction with those populations will increase.

### 3.3 Reducing Inbreeding Depression by Exchanging Genes

**FIGURE 3:**
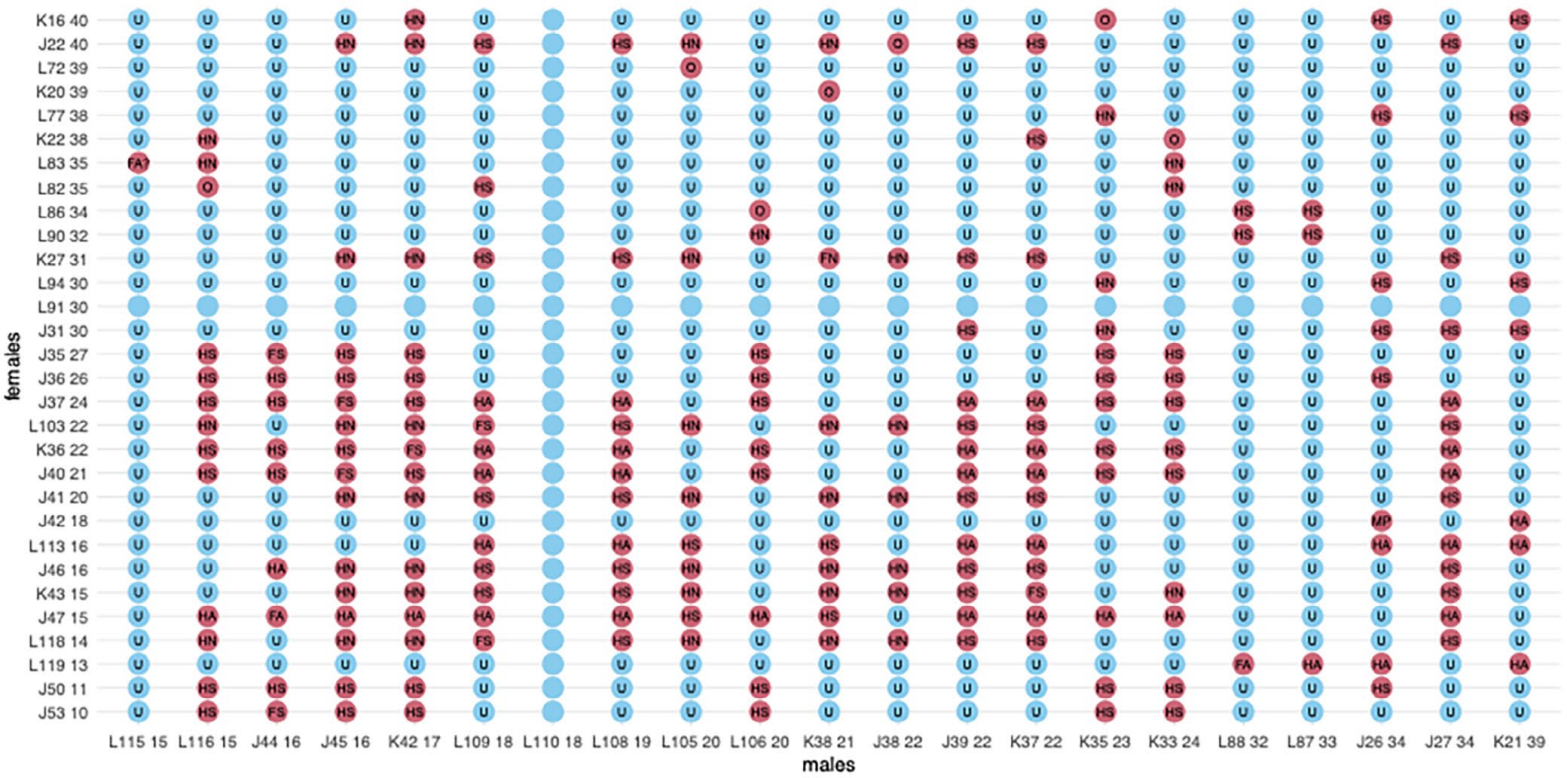
Relationships among breeding‐age females and males in the SRKW population. Relationships more distant than two generations are reported as unknown (light shading; U). Individuals with no genetic data are indicated by light shading only. First or second‐degree relationships (dark shading) are indicated as full sibling (FS), half‐sibling (HS), parent‐offspring (MP), full avuncular (FA), half‐avuncular (HA), and half‐niece/nephew (HN). The age of each individual (as of 2025) follows their identifier.

SRKW are more inbred and less genetically diverse than other NE Pacific killer whales, in part due to a greater historical decline from captures and other threats (NMFS 2008; Kardos et al. 2023). Inbreeding depression in SRKW is pronounced. Based on a model linking genomic estimates of inbreeding with observed SRKW demography, the most inbred individuals are estimated to survive to the end of their reproductive years at less than half the rate of the least inbred individuals (Kardos et al. 2023). Matings are known to increasingly occur between close relatives (Ford et al. 2018; Kardos et al. 2023). There are only 30 reproductive‐age females (many near the end of their reproductive years) and 21 reproductive‐age males, and the effective population size (*N*_e_) is only ~27. Most of the reproductive‐age individuals are related to each other, especially among younger animals (Figure 3). Inbreeding is a plausible explanation for the reduced population growth rate compared to other populations, and is a major factor in the population's predicted decline (Kardos et al. 2023).

The only realistic scenario to reduce inbreeding depression in the SRKW population is outbreeding with another population. Such “genetic rescue” (whether assisted or natural) is well documented in other species (Whiteley et al. 2015; Clarke et al. 2024), including large mammals such as panthers (Onorato et al. 2024) and wolves (Åkesson et al. 2016). A recent study of small, low latitude killer whale populations also found that interbreeding between genetically differentiated groups is important for limiting the effects of inbreeding (Reeves et al. 2025). Globally common deleterious genetic variants have also been found to be selectively purged in some small killer whale populations (Foote et al. 2025), further supporting the importance of genetics in this species' viability.

Genetic rescue of SRKW may be complicated due to learned behaviors and a culture that appears to limit outbreeding (Riesch et al. 2012), but the finding that other killer whale populations maintain diversity through interbreeding suggests that it may be possible. One population that SRKW individuals might interbreed with is the ecologically and behaviorally similar NRKW. Interbreeding between the two populations has not been documented, but is possible based on their overlapping range, increasing use of shared habitat, and high degree of genetic and ecological similarity. In addition to the global genomic patterns indicative of past gene flow discussed above, genetic evidence also suggests SRKW diverged from other fish‐eating populations only several hundred years ago (Kardos et al. 2023). There is evidence of low level historical (Hoelzel et al. 2007) and perhaps even recent (Pilot et al. 2010) gene flow between SRKW and SARKW. The SRKW and NRKW eat the same prey and utilize the same habitats, sometimes at nearly the same times. Despite these similarities, there is also evidence of small scale habitat partitioning between the populations; however (Emmons et al. 2021; Pilkington et al. 2023). Whether these behavioral barriers to interbreeding break down over time may well determine the future of the SRKW population.

## 4 Imagining Possible Futures for SRKW

In this section, we outline several hypothetical futures for SRKW, based on the preceding discussion. Our goal is not to predict what will happen or to list every possible scenario, but to imagine a range of scenarios that illustrate the importance of interactions with other killer whale populations. Considering these scenarios may also help motivate future research

1. *Current recovery scenario*—Recovery efforts targeted at SRKW are fully successful. These include substantial increases in prey that preferentially benefit SRKW, and major reductions in other environmental threats. The population remains genetically isolated and inbred, but experiences growth over a period of multiple decades due to improving environmental conditions. This scenario aligns with the assumptions and goals of the current United States and Canadian recovery plans.
2. *Replacement scenario*—SRKW remain genetically isolated and continue to decline due to inbreeding, even as recovery actions succeed in increasing prey and reducing threats. NRKW continue to grow and extend their range southward on the coast and in the Salish Sea, increasingly occupying the former SRKW range. By 2100, the SRKW lineage has mostly died out, but fish‐eating killer whales remain common in the Salish Sea and Washington coastal areas—as the descendants of NRKW or offshores.
3. *Extinction scenario*—Salmon abundance decreases and other environmental threats grow worse. SRKW remain isolated and continue to decline. NRKW and offshores also decline, or their distribution shifts northward. By 2100, the SRKW lineage has ceased to exist, and fish‐eating killer whales are rare or absent in the Salish Sea and Washington coastal areas.
4. *Genetic rescue scenario*—The ranges of SRKW and NRKW continue to overlap and expand in space and time such that occasional matings occur between the two populations, or between SRKW and offshores. The offspring of SRKW females and NRKW or offshore males have improved survival due to outbreeding. As a result, the SRKW population grows and persists as a distinct population.
5. *Population fusion scenario*—Interbreeding between SRKW and NRKW (or perhaps offshores) becomes common, such that the lineages effectively merge into a single meta‐population. SRKW matrilines become pods within a larger SR‐NRKW population.

## 5 Opportunities for New Research

We believe the results obtained over the past several decades, summarized above, illustrate the importance of considering interactions with other populations when planning for SRKW recovery. Much remains unknown, however, and our understanding would benefit from additional research. Below, we outline important research questions related to population interactions:

1. Inbreeding depression and prey abundance have both been found to be correlated with SRKW demographic rates, but their relative importance has not been determined. Additional population modeling is needed to evaluate the plausibility that prey increases alone will be an effective conservation program if the population remains inbred.
2. In recent years, efforts have been made to increase Chinook salmon prey availability through harvest reductions and increased hatchery releases. These efforts have focused on salmon stocks thought to be important to SRKW, but their effects on other killer whale populations are unknown. Additional evaluations on whether it is biologically or logistically feasible to increase prey preferentially for SRKW compared to NRKW and SARKW would be useful.
3. Previous modeling results have hinted that NRKW population growth may depress SRKW birth rates (Nelson et al. 2024). It would be useful to build on these results to develop joint population models that include SRKW, NRKW, and SARKW in order to evaluate density dependent effects across each of these populations.
4. Parentage analysis has been useful for studying mating patterns within and among killer whale populations (Barrett‐Lennard 2000; Pilot et al. 2010; Ford et al. 2018). This work could be expanded to include more thorough genetic monitoring of multiple killer whale populations so that any current interbreeding events would be detected. Especially when populations remain small, recurring gene flow may be needed to maintain fitness (Hedrick et al. 2014; West et al. 2025), so understanding its frequency is important.
5. More intensive and regular monitoring of SRKW, NRKW and offshore diets (both species and stocks) and behavioral interactions in the areas where they overlap, including the Washington coast and Strait of Georgia, would help to better evaluate competition for prey or space, and behaviors that either promote or restrict opportunities for interbreeding. This could include evaluating the possibility of using artificial calls or behavioral cues to increase social or reproductive interactions between SRKW and other populations.
6. SRKW recovery goals (NMFS 2008) were developed for a single population and may not fully consider our current understanding of the interactions among populations. Evaluating the recovery metrics to better incorporate the dynamics and trajectories of other fish‐eating killer whales in the NE Pacific would be useful. This evaluation might benefit from comparisons to recovery strategies for other large, widely distributed predators with interacting populations, such as the North American Grizzly Bear (USFWS 2024) and Gray Wolf (USFWS 2023).

## 6 Conclusions

Populations rarely persist for long in isolation from conspecific populations. Isolated populations, particularly if they are small, are vulnerable to inbreeding depression, loss of diversity, and extinction due to random events (Soulé 1986). SRKW are a distinct population, but they are not isolated. Decades of research have shown that they interact with other killer whale populations through shared prey and habitat, and, over longer time periods, potential interbreeding. Competition with other populations for shared prey or habitat may limit SRKW population growth, while interbreeding, if it occurs, may promote it. The prospects for SRKW recovery will therefore benefit from a continued focus on threat mitigation, combined with research to better understand actual and potential interactions with other killer whale populations.

## Author Contributions

**Michael J. Ford:** conceptualization (lead), writing – original draft (lead). **Eric J. Ward:** conceptualization (equal), writing – review and editing (equal). **Marty Kardos:** conceptualization (equal), writing – review and editing (equal). **Kim M. Parsons:** conceptualization (equal), writing – review and editing (equal). **Candice Emmons:** conceptualization (equal), writing – review and editing (equal). **M. Bradley Hanson:** conceptualization (equal), writing – review and editing (equal).

## Conflicts of Interest

The authors declare no conflicts of interest.

## Data Availability

The authors have nothing to report.
